# Ambivalent changes in the correlation of energy consumption and economic growth in the member states of the European Union (2010–2019)

**DOI:** 10.1016/j.heliyon.2023.e14550

**Published:** 2023-03-13

**Authors:** Török László

**Affiliations:** Department of Engineering Management and Enterprise, Industrial Process Management Institute, Faculty of Engineering, University of Debrecen, Ótemető u.2-4, 4032, Debrecen, Hungary

**Keywords:** Energy economy, Energy consumption, Economic growth, European Union, Energy-growth nexus, Sustainable energy consumption, Correlation, Cluster analysis

## Abstract

Energy consumption efficiency will be of paramount importance in 2023 due to the first global energy crisis experienced worldwide. The central question of this paper is how energy consumption and economic growth have been related to each other and how they have interacted in time and space in the European Union. Correlation calculations are used to show the relationship between the two indicators per Member State, and hierarchical cluster analysis is applied to the results. The analysis is based on data regarding the European Union Member States for the period of 2010–2019. The results show that there was no robust, strong correlation between the GDP and the energy consumption of the member countries. Another result shows that in the Member States where energy consumption fell significantly, the reduction did not have a negative impact on the economic growth. Finally, the study highlights the methodological shortcomings of studies on the correlation between energy consumption and economic growth.

## 1 Introduction

The study of the relationship between energy consumption and economic growth has been of interest to energy economists for decades, although the purpose and the methodology have undergone significant changes over the past few decades. Examining the correlation between the two indicators is relevant for energy economy researchers because it has been proven that it is impossible to realize the economic output of infinite value from the available non-infinite amount of energy. Consequently, the specialists have started to look for the methods by which the finite amount of energy sources can be utilized with increasing efficiency. The result of increasing energy efficiency will mean that more economic output can be realized with less energy.

The current mainstream ecological paradigm fails to recognize that energy consumption is governed by the fundamental laws of physics. This means that the economy's energy consumption falls under the scope of the laws of thermodynamics. The energy consumed to produce GDP is not lost, it is transformed, one of the consequences of which is global warming. This also means that the production of GDP/as an economic process/has an impact on the environment, because in this process there is a final consumption of energy that humans cannot replace with anything else. Therefore the difficulties of replacing energy sources undermine the stability of the economic cycles in our national economies.

Nowadays, securing energy consumption has become, besides human resources, the most important factor in achieving economic growth in the European Union and all national economies in the world.

The relationships between energy consumption and gross value of production (GDP), industrial production value and national income have been investigated by macroeconomists using mathematical-statistical methods. Methods may include trend calculations, correlation analyses, and elasticity analyses. These methods are of limited use in describing the nature and closeness of these relationships and in making reliable forecasts. One of the questions that researchers using these methods seek to answer is whether there is a point in economic output where economic growth no longer generates additional energy consumption. Another very important question is the direction of causality in the relationship between energy consumption and the gross value of production. Specifically, the question is whether an increase in energy consumption leads to an increase in GDP, or vice versa, with higher GDP production leading to more robust energy use. A large and growing body of literature has examined the relationship between these two indicators.

Several relevant studies argue that human prosperity is based on natural resources, such as energy, water, land, and other raw materials. At the same time, economic growth is leading to ever more intensive use of these resources, causing severe environmental pressures. In other words, resources and resource usage (e.g. energy consumption) theoretically provide the link between economic performance and environmental pressures [1,2].

A large body of published studies describes and accepts the fact that the energy consumption and the output of an economy are closely related, but the direction and strength of the relationship between the factors are subject to debate.

The paper seeks to answer the question of how the robust economic growth in the European Union between 2010 and 2019 affected the EU's energy consumption and whether a feedback hypothesis between the two indicators can be detected.

The hypotheses of the study are:

Hypothesis 1 There is a significant, strong positive correlation between energy consumption and economic growth in the European Union between 2010 and 2019.

Hypothesis 2 In EU Member States where the energy consumption significantly influenced the economic growth, a decrease in the energy consumption has a negative impact on the economic growth.

The formulation of the two hypotheses is based on the following:

In recent times, the attention of energy economics has focused primarily on providing the energy necessary for the operation of the economy. This significant attention can be attributed to the fact that the outbreak of the Russian-Ukrainian war made the energy supply of the European Union uncertain.

As a result, the unstable energy supply endangers the economic growth of the European Union.

Economic growth (which is shown by the increase in GDP) means that the welfare of the members of a national economy increases, and their opportunities for consumption and satisfaction of needs expand. (This finding is disputed by many researchers, but this study does not intend to take sides in this debate). Energy consumption and GDP are closely related, as energy largely determines the economic development of the countries.

Based on the previous general rule, an increase in GDP is associated with an increase in energy demand. At the same time, this correlation can be slightly influenced by the improvement of energy efficiency or the structural changes in individual economies from time to time. During such structural changes, for example, energy-intensive industrial sectors are pushed back and replaced by less energy-intensive services. However, the service sector currently represents around three-quarters of the GDP in the developed countries, which means that this sector is a huge consumer of energy. It should also be noted that the energy demand of the service sector is relatively lower, but empirical evidence shows that the energy demand for services also increases, for example, with the advancement of digitalization. It can be reasonably stated that a significant amount of literature has been published in recent decades on the relationship between the two indicators (energy consumption and GDP). These studies prove that there is a demonstrable relationship between the two indicators. In the following literature section, this study presents several papers that examine this correlation.

### 1.1 Literature review

A review of the literature shows that ecological and energy economists agree on the primary role of energy in economic growth. Over the past decade, a large number of publications have been published on the subject, although the research results are still not uniform. The differences are mainly due to the use of different econometric methods and periods. The heterogeneity of national states with different consumption patterns, climates, economic development, and all the factors that influence the two variables mentioned above, also contributes to the differences in results [3]. Ecology and energy economists consider energy and energy use to be the main drivers of economic growth. Indeed, some radical experts believe that energy use is the sole measure of economic development [4]. Other experts explain their theories in terms of the important role of energy use in production. They argue that there is no economic activity that does not require energy, based on the simple fact that production is a work process and that work involves energy inputs [5,6]. One of the researchers points out that the first, second, and third industrial revolutions in human history were the main sources of rapid progress. Many of the then newly discovered technological advances were linked to some hitherto unused source of energy (coal - steam engines; oil - internal combustion engines; nuclear energy - cheap electricity) [7]. There is a strong positive relationship not only between energy use and economic growth but also between energy use and the human development index (HDI) [8]. A widely cited paper examines the relationship between the two variables analyzed in this study in OECD countries in the period 1980–2010. The research results show that there is a significant relationship between energy consumption and economic growth in OECD countries over the period under study [9]. Researchers examined the relationship between the two variables with regard to the economies of African countries. Another study examines the causal relationship between energy consumption and real GDP in fourteen MENA (Middle East and North Africa) countries over the period 1987–2019. The results show that there is a multidirectional causal relationship between the two variables [10]. One study finds evidence of the extensive nature of energy consumption. The authors show that the impact of energy consumption on GDP is more intense in non-OECD countries than in Organisation for Economic Co-operation and Development (OECD) countries [11]. Hannesson (2009) argues in his study that there is a positive but not proportional relationship between GDP and energy consumption. There are indications that energy use growth decreases with GDP per capita for any given increase in GDP [12]. One study examines energy consumption and economic growth in developing and developed countries. In the case of developed countries, researchers find less evidence of the closeness of the relationship between the two examined variables than in the case of developing countries [13]. A study examines the regional emergence of the relationship between the two indicators in China. The authors find that there is significant spatial agglomeration in the spatial distribution of regional economic growth and energy consumption, with a core-periphery model emerging, with the eastern region as the core [14]. A study reaches a robustly different conclusion from the Chinese one when it expands its investigation to several US states. The referenced paper analyzes the relationship between the two variables examined in this study in the 47 states of the United States between 1997 and 2009. The test shows that there is a long-term equilibrium relationship between the two variables [15].

Most of the literature examines the relationship between the two examined variables and CO2 emissions. A study has shown that CO2 emissions increase when the economies of Europe and Central Asia are booming. Rapid growth in energy consumption and foreign direct investment have an impact on CO2 emissions, as foreign investment increases both production and energy consumption [16]. Most of the existing studies use countries as observation units for the gross domestic product (GDP) and energy consumption. This perspective makes it difficult to interpret and generalize the results because the countries are at different stages of development, they are very different from each other in terms of economic development, culture, technology, and other segments [17]. According to a study by a research group, there is mixed evidence on the direction of the cause-effect relationship between the two investigated variables in the short term. In the medium and long term, however, the majority of the subgroups only have a two-way causality [18]. A study analyzes the relationship between energy production and GDP in several countries. The shift in the composition of energy production is not uniform in the examined countries. The policy recommendations made in the study were based on the relationship between the two analyzed variables in the period under review. As countries step up their efforts to modernize electricity grids and move towards more environmentally friendly forms of production, policy recommendations should be reevaluated at each time frame [19]. Researchers use a threshold regression technique to find out whether there is a threshold value of GDP for energy consumption. The results of the study highlight the existence of a GDP threshold. According to this, the effect of GDP on energy consumption and the causal direction of the increase in consumption depend on the initial value of GDP [20]. According to a study on trends in energy consumption, modified ordinary least squares (FMOLS) estimates show that fossil fuel consumption contributes to emitting carbon dioxide. Contrary to the previous statement, the increase in the use of renewable energy and financial development reduce carbon dioxide emissions [21]. A study uses panel data and a new model to examine the relationship between the two variables analyzed in this paper. The main results suggest that the elasticity between the two indicators is much smaller than in other empirical studies [22]. The results of a panel data model research report that FDI and energy consumption together can significantly increase GDP output in a national economy. However, poverty, interest rates, and inflation are negatively and significantly related to economic modernization in developing countries in the Asian region [23].

## 2 Material and methodology

This article is closely related to energy economics, since its central question is the relationship between the two variables examined, as well as their interaction in time and space. Correlation and causality between the two indicators are an important part of energy economics research and are the main research focus of this study.

### 2.1 Databases

GDP data from Eurostat [24] and energy consumption data from the European Commission [25] are used in this study to examine the data for the period 2010–2019. The economic growth outcome is highly dependent on the base year, which is 2010 in this study. The choice of 2010 as the base year is explained by the fact that in the first decade of the new millennium, many countries experienced significant economic overheating, followed by a significant downturn in 2008/2009. The economic downturn was a consequence of the subprime crisis. By 2010, however, the majority of the EU Member States had overcome the worst of the shock and were beginning to recover. An unprecedented period of economic growth began between 2010 and 2019, which lasted until the onset of the global pandemic in 2020, making the years 2010–2019 an appropriate period for the study's focus. GDP is the basis for the analysis of economic growth. GDP seems to be a better choice than GNP because energy consumption is more related to the goods and services produced in the national economy.

### 2.2 Methodology

The two main indicators analyzed are the final energy consumption and the economic growth in the EU Member States. The first indicator is the amount of energy delivered to the final consumer at the point of consumption. The latter indicator uses GDP measured in Purchasing Power Standard (PPS) for comparability.

The study calculates the comparison of economic growth (GDP change) between Member States using Purchasing Power Standard (PPS) units. This method can be used to deal with differences in price levels between member countries. The GDP values adjusted for price level differences can give a realistic picture of the actual economic growth of the member countries. Studies that do not use the PPS (but, for example, current price GDP data) to examine economic growth are methodologically flawed and therefore their results and conclusions are flawed. Studies using such incorrect methods are referred to in the discussion section of this paper.

The methodological basis of the analysis is a combined application of correlation analysis and hierarchical cluster analysis. Correlation refers to the mutual relationship between indicators. The two indicators in this study are the energy consumption and the GDP of the EU member states.

An Excel program calculates the correlation coefficient using the mean data in Table 1, Table 2. However, this is only the first procedure in the analysis of the relationship between the indicators. In the second procedure, a hierarchical cluster analysis is performed with the calculated correlation coefficients. These two procedures are often used by researchers for econometric analyses [[26], [27], [28], [29]]. This combined method is also used in a subsequent study. The selection of indicators is a fundamental technique for reducing the dimensionality problem of data mining tasks. Traditional feature selection algorithms cannot be scaled widely and over a large area. The referenced article proposes a new method to solve the dimensionality problem, where clustering is integrated with correlation coefficients to construct subsets with appropriate properties [30].

**Table 1:** Gross domestic product (GDP) in the member states of the European Union, (in billion PPS euros), 2010–2019.

Country	2010	2011	2012	2013	2014	2015	2016	2017	2018	2019	Index ‘19/'10
BE	329	331	339	355	363	374	383	394	406	424	128.9
BG	84	86	89	89	92	95	99	104	108	116	138.1
CZ	221	214	217	233	244	257	268	279	292	311	140.7
DK	181	175	180	189	193	200	209	217	225	232	128.2
DE	2411	2521	2579	2678	2796	2796	2896	2994	3071	3146	130.5
EE	22	23	24	26	28	28	29	30	33	34	154.5
IE	149	148	151	162	170	233	245	257	280	293	196.6
EL	235	226	216	217	217	208	212	214	222	221	94.0
ES	1117	1121	1125	1130	1163	1165	1213	1260	1289	1337	119.7
FR	1767	1782	1815	1908	1938	1954	2001	2046	2116	2245	127.1
HR	65	65	67	67	68	70	73	75	78	84	129.2
IT	1577	1547	1861	1587	1602	1611	1670	1729	1760	1803	114.3
CY	21	20	20	19	19	19	21	22	24	25	119.0
LV	28	31	33	34	35	36	37	38	40	42	150.0
LT	47	51	55	58	60	60	63	65	68	73	155.3
LU	35	35	36	38	41	44	45	46	48	49	140.0
HU	165	168	169	176	184	190	194	198	210	223	135.2
MT	9	9	9	10	10	12	13	13	14	16	177.7
NL	566	542	545	595	606	612	630	648	675	696	123.0
AT	266	271	279	297	303	310	319	328	342	351	132.0
PL	607	630	660	688	715	734	759	783	822	873	143.8
PT	218	205	205	216	223	221	227	233	240	253	117.4
RO	260	260	272	289	302	308	337	365	388	420	161.5
SI	43	43	44	44	47	47	50	52	55	58	134.9
SK	103	102	105	110	114	117	116	115	121	119	115.5
FI	158	157	159	164	165	167	173	179	185	189	119.6
SE	300	292	297	319	327	346	354	362	372	382	127.3
**EU27**	**10,982**	**11,055**	**11,264**	**11,698**	**12,024**	**12,215**	**12,636**	**13,047**	**13,484**	**14,015**	**127.6**

Shapiro-Wilk test results (GDP): P-value: 0.06091, W: 0.9278, Sample size (n): 27, Average ($\bar{x}$): 35.3333, Median: 30.5, Sample Standard Deviation (S): 20.9558, Sum of Squares: 11417.82, b: 102.9223, Skewness: 1.0537.

**Table 2:** Gross inland consumption in Mtoe (Million tons of oil equivalent), 2010–2019.

Country	2010	2011	2012	2013	2014	2015	2016	2017	2018	2019	Index ‘19/'10
BE	61	57	54	56	54	54	56	56	55	56	92
BG	18	19	18	17	18	19	18	19	19	19	106
CZ	45	44	43	43	42	42	42	43	44	43	96
DK	20	19	18	18	17	17	18	18	18	17	85
DE	338	321	324	331	317	318	320	321	315	308	91
EE	6	6	5	6	6	5	6	6	6	5	83
IE	15	14	14	13	14	14	15	15	15	15	100
EL	28	28	27	24	24	24	24	24	24	24	86
ES	130	130	129	121	118	123	124	130	130	127	98
FR	270	264	265	266	256	260	255	256	254	251	93
HR	9	9	9	9	8	9	9	9	9	9	100
IT	177	171	164	158	150	156	154	160	157	155	88
CY	3	3	3	2	2	2	2	3	3	3	100
LV	5	4	5	4	4	4	4	5	5	5	100
LT	7	7	7	7	7	7	7	8	8	8	114
LU	5	5	4	4	4	4	4	4	5	5	100
HU	27	26	25	24	24	25	26	27	27	27	100
MT	1	1	1	1	1	1	1	1	1	1	100
NL	86	81	81	79	75	76	78	79	78	76	88
AT	35	34	34	34	33	34	34	35	34	35	100
PL	102	102	98	99	95	96	101	105	110	106	104
PT	24	24	22	22	23	24	24	25	24	24	100
RO	35	36	35	32	32	32	32	34	34	33	94
SI	7	7	7	7	7	7	7	7	7	7	100
SK	18	17	17	17	16	16	16	17	17	17	94
FI	37	35	34	34	34	33	34	34	35	34	92
SE	50	51	51	50	49	47	49	50	51	50	100
**EU27**	**1559**	**1514**	**1494**	**1479**	**1428**	**1448**	**1460**	**1491**	**1481**	**1458**	**93.5**

Shapiro-Wilk test results (Gross inland consumption): P-value: 0.07013, W: 0.9303, Sample size (n): 27, Average ($\bar{x}$): 96.4296, Median: 100, Sample Standard Deviation (S): 6.9424, Sum of Squares: 1253.1363, b: 34.143, Skewness: 0.09898.

The multivariate statistical method of cluster analysis is used in this study to determine homogeneity across EU member states. Cluster analysis is conducted to classify the EU Member States under study into clusters based on the degree of association between the two indicators. The Between Groups method is used for clustering. Before clustering, however, it is necessary to check whether the values of the indicators are normally distributed. For this purpose, a Shapiro-Wilk test is performed on GDP, energy use, and correlation coefficients. Finally, the study uses the so-called partitioning methodology to determine which cluster a given eurozone member state belongs to, depending on how strong the relationship is between the two variables of the analyzed member state.

**Fig. 1:**
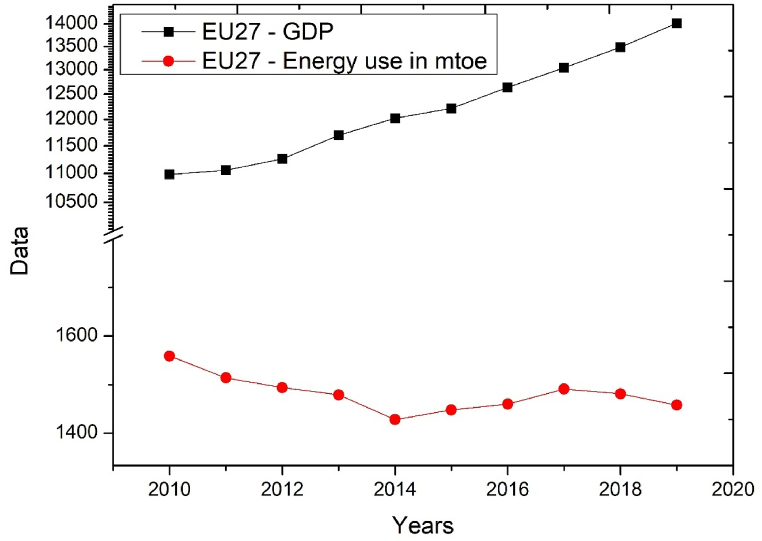
The results belonging to EU 27 Member States by year.

Fig. 1 illustrates the separation of EU economic growth and energy consumption between 2010 and 2019.

## 3 Results

Results are presented in three subsections in the following order. First, the results of the time series analysis of GDP change are presented, followed by the results of the energy consumption time series analysis. Finally, the central question of the paper is addressed, where the results of the analysis of the relationship between the two indicators are presented.

### 3.1 Results of the time series analysis of GDP change

The European Union economy grew by an average of 2.45% per year in absolute terms, with an annual growth rate of €3033 billion in 2019 compared to 2010.

The data in Table 1 show that Greece is the only country whose GDP decreased compared to the 2010 base period. The explanation for this decline is that Greece suffered the most severe loss of growth potential. After a continuous decline in potential output, in 2019 it only reached the 2008 level. The fall in productivity was the main factor in the unprecedented GDP decline in the European Union.

Italy, Slovakia, Portugal, Cyprus, and Spain are the countries where GDP growth was between plus 0 and 20%. In the Netherlands, France, Sweden, Denmark, Belgium, Croatia, Germany, Austria, Slovenia, Hungary, Bulgaria, and Luxembourg, economic growth was between plus 20 and 40%. GDP growth in Poland, Lithuania, Estonia, and Lithuania was between plus 40 and 60%.

Romania and Malta grew by plus 60–80%, and Ireland by 96.6%. These three countries had the most dynamic growth performance in the European Union between 2010 and 2019. Romania's domestic consumption and investment, Ireland's extremely high investment rate, and Malta's high investment rate were the main drivers of economic growth [31].

### 3.2 Results of time series analysis of energy consumption

Energy consumption in the EU economy decreased by an average of 0.7% per year in absolute terms by 101 Mtoe in 2019 compared to 2010, with a value of 1458 Mtoe in 2019. On average, 1 Mtoe of energy consumption in the EU generated €9.61 billion of GDP. The histogram of energy use in 2019 and the data in Table 2 show that Estonia, Denmark, Greece, Italy, Greece, and the Netherlands reduced their energy use by between 10 and 20% compared to 2010 respectively. Germany, Belgium, Finland, France, Germany, Romania, Slovakia, the Czech Republic, and Spain reduced their energy use by between 0 and 10%. Ireland, Croatia, Cyprus, Lithuania, Luxembourg, Hungary, Malta, Austria, Portugal, Slovenia, Sweden, and Slovenia did not change their energy use. Poland and Bulgaria increased their energy use by 3.9% and 5.6% respectively. The largest increase in energy use was in Latvia, one of the smallest economies in the European Union, with 14.3%.

More relevant correlations emerge when comparing the GDP per 1 Mtoe of energy use in 2019 in the Member States. From the data in Table 1, Table 2, the following ranking of energy use efficiency per 1 Mtoe is established. There are nine Member States with the most efficient energy use. In these Member States, more than €10 billion of GDP were generated with 1 Mtoe of energy use. Ireland, Malta, Denmark, Denmark, Romania, Italy, Portugal, Spain, Germany, and Austria lead the efficiency ranking. The previous cluster in energy efficiency is followed by countries that produced between €9 and €10 billion GDP with 1 Mtoe of energy. These countries are Luxembourg, Croatia, Greece, the Netherlands, and Latvia, respectively. In terms of energy efficiency, the third cluster is made up of countries with a GDP of €8–9 billion with 1 Mtoe of energy. These countries are France, Lithuania, Cyprus, Hungary, Poland, and Slovenia, respectively. The fourth cluster is made up of the least energy-efficient Member States, Sweden, Belgium, the Czech Republic, and Slovakia are the most energy-inefficient countries in the European Union, with Estonia, Bulgaria, and Finland producing the least GDP per unit of energy used.

The efficiency of energy use in the European Union compared to the US is that its productivity is 13.5% better. This figure can be demonstrated by the fact that while in the European Union, 1 Mtoe of energy use produced €9.61 billion of GDP, in the US it was US$ 9.62 billion in 2019, or €8.47 billion [32,33]. This result is confirmed by another study, which concludes that Europe is the region with the lowest primary energy intensity per unit of GDP at purchasing power parity. In other words, Europe is relatively efficient in converting energy into gross domestic product [34].

### 3.3 Results of the analysis of the correlation between GDP and energy consumption

Correlation calculation resulted in the following correlation coefficients (r): Belgium: −0.3507, Bulgaria: 0.5373, Czech Republic: −0.2486, Denmark: −0.5204, Germany: −0.8919, Estonia: −0.1656, Ireland: 0.6172, Greece: 0.6887, Spain: 0.1915, France: −0.8970, Croatia: 0.1799, Italy: −0.2536, Cyprus: 0.6123, Lithuania: 0.2023, Latvia: 0.7678, Luxembourg: 0.0079, Hungary: 0.4363, Malta: 0.6425, Netherlands: −0.6005, Austria: 0.1738, Poland: 0.5359, Portugal: 0.4945, Romania: −0.3168, Slovenia: −0.3519, Slovakia: −0.5055, Finland: −0.2656, Sweden: −0.2427.

Test Results (Correlation Coefficients): P-value: 0.1008, W: 0.9367, Sample size (n): 27, Average ($\bar{x}$): 0.021, Median: 0.0079, Sample Standard Deviation (S): 0.4937, Sum of Squares: 6.3363, b: 2.4362, Skewness: −0.09072.

**Fig. 2:**
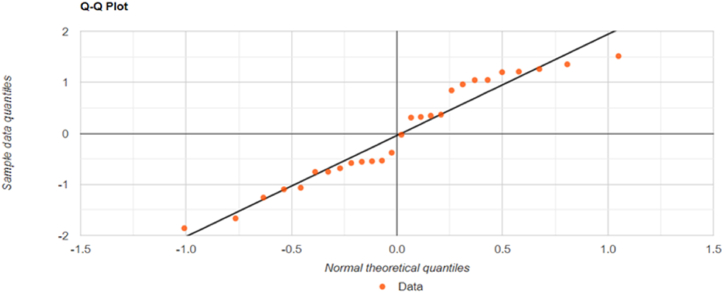
The standard deviation of the correlation coefficients between GDP and energy consumption of the European Union Member States.

Fig. 2 illustrates that the correlation data included in the study follows a normal distribution.

**Fig. 3:**
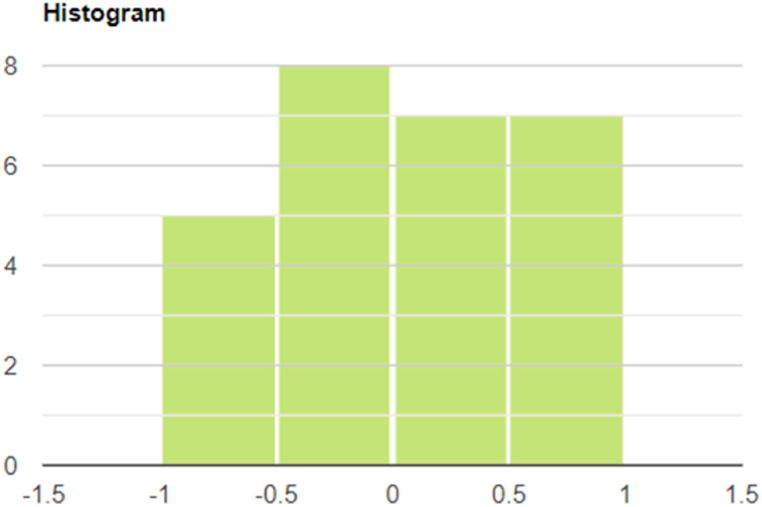
Clusters of EU Member States by the correlation between GDP and energy consumption.

Fig. 3 illustrates how many EU member states were included in each cluster based on the strength of the correlation between the two variables. The EU member states within some of the clusters shown in this figure are similar to each other according to the dimension of the relationship between the two variables examined in the study, and they differ from the member states in the other clusters along this dimension.

The correlation methodology is used to calculate the correlation coefficients of the member countries based on time series data on changes in GDP and energy consumption. Based on its values, the study classifies the EU Member States into four clusters.

The first cluster (interval: −1.0; −0.5) consists of five Member States, Germany, France, the Netherlands, Denmark, and Slovakia, respectively. For the countries listed, there is a strong negative inverse relationship between GDP and energy use. The negative inverse relationship between the indicators means that these Member States experienced a significant increase in GDP while their energy use decreased significantly. The average GDP growth in these Member States was 25.3%, while the average energy use was - 9.6%. From the data in Table 1, Table 2 and the values of the correlation coefficients with negative signs, it is clear that the year-on-year improvement in the energy efficiency of these five Member States is negatively correlated with the specific values of GDP, which increased dynamically over the period under review.

The second cluster (interval: −0.5; 0) includes eight Member States, Slovenia, Belgium, Romania, Finland, Italy, the Czech Republic, Sweden, and Estonia, respectively. In the countries listed, there is a weak, negative, inverse relationship between GDP and energy use. These Member States also experienced a moderate increase in GDP (34.6% on average), but a much smaller decrease in energy use (- 6.9%) than the previous cluster. Although the negative inverse relationship can be observed in this cluster, this explains why the relationship between GDP and energy use is significantly weaker than in the previous cluster.

The third cluster (interval: 0; 0.5) includes seven Member States, Portugal, Belgium, Romania, Finland, Italy, the Czech Republic, and Sweden, respectively. The sign of the correlation changes concerning the member countries listed with a weak, positive, linear relationship between GDP growth and energy use. The change in the direction of the correlation is explained by the fact that these Member States experienced moderate GDP growth (27.7%) but no change in energy use.

The fourth cluster (interval: 0.5; 1) includes seven Member States, Latvia, Greece, Malta, Ireland, Cyprus, Poland, and Bulgaria, respectively. For the listed Member States, there is a strong, positive, linear relationship between GDP growth and energy use. The strength of the relationship between the two indicators is that the average GDP growth of the seven listed Member States is significantly higher than the average GDP growth of the three previously clustered Member States. The growth rate is 46.4%. The robust economic growth was also accompanied by a +1.4% increase in energy consumption for this group. The combination of the highest GDP growth rate and the increase in energy consumption resulted in a strong positive correlation with a linear correlation for the seven EU Member States in the cluster.

## 4 Discussion

This paper empirically investigates the causal relationship between energy consumption and GDP for the 27 EU Member States from 2010 to 2019. When examining the relationship between GDP change and energy consumption, a first approach would be to expect these two indicators to move together and in the same direction. A co-variation would mean that if the GDP of an economy increases, its energy consumption should also increase. In such a case, the increase in GDP would be the causal factor (i.e. the independent variable) and the increase in energy consumption would be the cause (i.e. the dependent variable).

However, the results of the present study do not support the causal relationship described in the previous sentence. Indeed, the results of the study show that there is no robust, very strong correlation between GDP at PPS prices and energy consumption in the EU Member States, because GDP grew by 27.6%, while energy consumption fell by 6.5% in 2019 compared to 2010.

In the continuation of this chapter, this paper first presents literature that concludes that increases in energy consumption are positively correlated with increases in GDP. Following the review of this literature, the paper presents studies that conclude that GDP growth is not correlated or associated with increases in energy consumption.

The first study cited finds empirical evidence and identifies a one-way causal relationship between economic growth and energy consumption and concludes that energy consumption may have had a large impact on economic growth in several Far East countries [35]. In another study, a causal link between oil consumption and economic growth is found for the most developed European countries between 1980 and 2007. In these countries, the state had to devote additional resources to subsidise oil prices and to ensure long-term and stable oil resources for the economy. In such countries, a decline in oil consumption due to various causes can lead to a decline in economic growth [36]. Research on OECD countries' data from 1960 to 2005 detected a strong correlation between the two indicators. The results of the fully adjusted ordinary least squares and dynamic approaches methodology show that in most cases there is a strong relationship between the two variables analyzed in this study in the countries studied [37]. In summary, one study finds that the dependence of GDP growth on energy consumption is strong in non-OECD countries where the extensive determinants of economic growth are important. In the future, decarbonization policies should consider the exogenous factors for growth in developing countries. The economic growth of these non-OECD countries involves increasing energy consumption, as these economies are the main energy consumers [11].

The results of the studies cited below show that there is no strong correlation between the two indicators, which means that the increase in energy consumption does not result in GDP growth.

The results of a study show a one-way cause-and-effect relationship between the two variables examined in this thesis. Due to unidirectionality, energy consumption has a positive effect on GDP. This suggests that a potential energy saving program could harm economic growth in the long run [38]. The results consistently show a strong one-way causal relationship between energy consumption and economic growth in Saudi Arabia. However, the increased GDP requires huge energy consumption in Saudi Arabia, which also means that the country has to follow a different export policy than other countries that also export energy [39]. The strength of the relationship between the two indicators is influenced by the income group in which the economy under study is placed in the global economy. The results of one study show that the causal relationship between the two variables examined in this thesis differs depending on the income group of the country concerned. For example, lower-income countries consume less energy compared to other groups. This fact suggests that these countries use more traditional production methods. Therefore, they do not use energy resources efficiently in the production process and therefore need proportionally higher energy consumption for their economic growth [40].

Finally, this paper refers to research which, in the opinion of the author of this paper, uses inappropriate methodologies to investigate the relationship between energy consumption and economic growth, and therefore reaches unsubstantiated results and conclusions.

This study considers economic output and its change in terms of PPS (Purchasing Power Standard, abbreviated as PPS). This is the purchasing power parity. The significance of the PPS value is that it eliminates differences in the price levels of the countries being compared (in this study, the European Union Member States). By using PPP, these indicators (e.g. GDP) are converted by experts into an artificial common currency. The converted value is called the Purchasing Power Standard (PPS). The use of a purchasing power standard allows for comparisons between member countries even if they use different currencies and have different price levels [41].

It follows from the previous definition that when comparing countries, it is advisable to use GDP expressed in PPS because, for example, GDP figures at current prices lead to erroneous results and conclusions.

According to the author of the present paper, the following studies [[42], [43], [44], [45], [46]], for example, incorrectly consider GDP developments at non-PPS prices and GDP changes. Often, researchers take GDP at current prices, but then GDP does not accurately reflect the real output of the economy due to changes in market prices. A method of calculation that filters out the effect of price changes are therefore needed to determine the true change in output. To filter out differences in price levels, PPS values can be used.

At the very end of the discussion chapter, the study deals with the question of what alternatives the European Union has to use other energy sources instead of traditional fossil energy carriers to create a sustainable energy economy during the global energy crisis. Such energy sources are renewable energies. Renewable energy sources are available in unlimited quantities, they are constantly regenerated and never run out. Since anyone can access these energy sources at any time, the energy source itself has no price. Renewable energy sources are the following: solar energy, biomass, wind energy, hydro energy, energy from sea waves, geothermal energy, and tidal energy.

International examples show that the hybrid use of renewable energy sources, complementary to each other and to fossil energy sources, can help stabilize the energy consumption of national economies. A study proves the effectiveness of the DTR (Dynamic Thermal Rating) system. The thesis proves that DTR improves the performance efficiency of existing, traditional energy systems by 30–50% with lower costs and shorter implementation times [47]. Because of the positive results, the DTR system has been widely used to improve the integration of renewable energy sources [48].

The following study proves another advantage of using the DTR system. The paper demonstrates that although the DTR system introduces additional line load, the reliability advantage of the power supply system far outweighs the disadvantage of increasing line unavailability [49].

A study also examines the flexible repair of networks. The paper applies network topology optimization to optimize line and rail switching to alleviate network congestion and improve network resilience. A dynamic thermal rating system is used to improve the rating of overhead lines. The battery storage system is used to time-shift wind energy use and avoid wind spillage. These methods are effective and proven, though they were only studied in isolation by specialists [50]. A study covers the information and communication technologies (ICT) used in the operation of energy networks and their cyber security limitations. ICT integrates infrastructures into the energy network with the help of technologies such as active distribution networks, smart cities, as well as dynamic line ratings, special protection systems and demand-side management programs. The use of these technologies has a positive and beneficial effect on the reliability and stability of energy systems. However, these infrastructures are naturally prone to failures and cybersecurity issues due to IoT standards, which can further compromise system reliability [51].

At the time of writing this study, the European Union is facing an unprecedented energy crisis. To alleviate the energy crisis, the use of renewable energy sources in a greater proportion and the importance of storing the produced energy has also come to the fore. Among the options for energy storage, the study highlights the battery energy storage system (BESS: Battery Energy Storage System). BESS is a technology solution that allows users to store energy from various sources such as solar, grid, and wind for later use. A study draws the attention of professionals to the fact that it is not optimal to install BESS systems without considering network topology and cooperation. Therefore, the paper proposes a method that optimally deploys BESS systems and determines their capacity in a two-part framework to minimize solar energy limitation [52]. A study draws the attention of specialists to the importance of optimization. The paper proposes a multi-objective framework that optimizes real-time line thermal rating and battery storage capacity enhancement against wind limitation, grid aging, and reliability. The results show that the maximum allowable temperature of the transmission line affects all three optimization parameters, but the battery efficiency only affects the wind restriction level [53].

## 5 Conclusion

This paper empirically investigates the relationship between energy consumption and GDP for the 27 EU Member States from 2010 to 2019. The calculations performed using correlation calculations and hierarchical cluster analysis methods do not confirm any of the hypotheses. The first hypothesis is not confirmed because the GDP of the EU Member States increased by 27.6% between 2010 and 2019, while the energy consumption of the EU Member States decreased by 6.5%. The relative decoupling of the two indicators occurred during the period under study because the EU GDP growth rate was positive, and the energy consumption rate was negative between 2010 and 2019. In absolute terms, the decoupling of energy use and GDP occurs when energy consumption (environmental pressure) does not increase and only economic output increases. However, this absolute decoupling is not confirmed by the GDP and energy consumption data for the EU Member States over the period under review.

The second hypothesis is not supported either, because in the EU Member States where energy consumption fell most significantly (the countries in the first cluster), the fall did not have a negative impact on the economic growth. On the contrary, despite an 8% decrease in energy consumption, Slovenia, Finland, Slovakia, Denmark, the Netherlands, Germany, and France all experienced significant increases in GDP at PPS prices over the period 2010–2019.

The importance of the study is that its results corroborate that the European Union made progress toward sustainable management over the examined period. This progress is confirmed by the study's finding that there was a decoupling between energy consumption and economic growth in the European Union, as energy consumption fell significantly over the period despite robust GDP growth.

The hierarchical cluster analysis shows that the EU countries are in significantly different positions in decoupling energy consumption from economic growth. Experts are encouraged by the fact that the Member States with a large industrial base (Germany, France, Denmark, and the Netherlands) achieved the most significant reduction in energy consumption. By contrast, the Member States with a bigger service sector (Ireland, Greece, Malta, Cyprus) did not realize any significant reduction in energy consumption over the period.

## Funding

This research received no specific support from the government, commercial, or non-profit sector funding agencies.

## Additional information

No additional information is available for this paper.

## Declaration of competing interest

The author declares that he has no known competing financial interests or personal relationships that appear to have influenced the work described in this study.
